# Effect of Laccase-Mediated Biopolymer Grafting on Kraft Pulp Fibers for Enhancing Paper’s Mechanical Properties

**DOI:** 10.3390/polym9110570

**Published:** 2017-11-02

**Authors:** Lourdes Ballinas-Casarrubias, Luis Villanueva-Solís, Carlos Espinoza-Hicks, Alejandro Camacho-Dávila, Hilda Amelia Piñón Castillo, Samuel B. Pérez, Eduardo Duarte Villa, Miguel De Dios Hernández, Guillermo González-Sánchez

**Affiliations:** 1 Facultad de Ciencias Químicas, Universidad Autónoma de Chihuahua, Circuito Universitario s/n, Campus Universitario No. 2, C.P. Chihuahua 31125, Chih., Mexico; a252283@uach.mx (L.V.-S.); jhicks@uach.mx (C.E.-H.); acamach@uach.mx (A.C.-D.); sperez@uach.mx (S.B.P.); 2 Centro de Investigación en Materiales Avanzados, Laboratorio Nacional de Nanotecnología, Miguel de Cervantes No. 120, Chihuahua 31109, Chih., Mexico; hilda.pinon@cimav.edu.mx (H.A.P.C.); guillermo.gonzalez@cimav.edu.mx (G.G.-S.); 3 Grupo COPAMEX, Ave. de la Juventud No. 280, San Nicolás de los Garza 66450, Mexico; eduardo.duarte@copamex.com (E.D.V.); miguel.dedios@copamex.com (M.D.D.H.)

**Keywords:** paper, biografting, cellulose, polyphenols, laccase

## Abstract

High-resistance paper was manufactured by laccase-grafting of carboxymethyl cellulose (CMC) and chitosan (CPX) on Kraft pulp fiber. The reaction was mediated in the presence of laccase by one of the following polyphenols in the presence of air: gallic acid (GA), vanillic acid (VA) and catechol (1,2–DHB). Enzyme was added at constant loading (24 kg ton^−1^), 1% pulp consistency, 0.005% CMC, pH = 6.3 ± 0.5 and 2 mM of mediator. CPX content was assessed at two levels (0% and 0.005%). Treated pulps were analyzed by different mechanical tests (ring crush, mullen, corrugating medium test (CMT) flat crush of corrugating medium test and tension). An improvement in these parameters was obtained by biopolymer coupling and selected mediator. When using GA, three parameters increased more than 40%, while ring crush increased 120%. For the case of VA, properties were enhanced from 74% to 88% when CPX was added. For 1,2–DHB, there was not found a statistically significant difference between the results in the presence of CPX. Scanning electron microscopy, confocal microscopy, FTIR and ^13^C NMR were used in all papers in order to evaluate grafting. Hence, it was possible to correlate polymerization with an improvement of paper’s mechanical properties.

## 1. Introduction

Process innovation relies on developing new products and technologies under green chemistry principles. Currently, industries are using enzymes for the implementation of eco-friendly technologies. As a result, they can overcome the limitations of physicochemical processes; especially in terms of the new regulation requirements. Enzymology has gained importance for new-product formulation [1,2,3]. Among the most-used enzymes in the paper industry are laccases (E.C. 1.10.3.2), which are produced naturally by many microorganisms, principally fungi. They catalyze the oxidation of substrates such as phenols and polyphenols with wide oxidative capability, due to their high redox potential [4]. Laccases require oxygen as co-substrate, rendering water as the only byproduct. For instance, they are able to oxidize specific phenolic end groups in lignin [5].

For the case of lignin-degrading organisms, laccases use low-molecular weight diffusible redox-mediator substrates [6]. Once oxidized, they are able to penetrate the lignin structure to interact with the non-phenolic groups. Mediators allow the oxidation of the complex substrate, which is not oxidized by the enzyme alone.

The discovery of synthetic mediators in the nineties [7] suggested a laccase mediator system (LMS) for delignification of cellulosic pulp. ABTS (2,2′-azino-bis(3-ethylbenzothiazoline-6-sulfonic acid) is one of the compounds used more extensively [8], even as a model substrate for many other enzymes, for example, horseradish peroxidases [9]. It generates a cationic radical, oxidizing organic compounds via an electron-transfer (ET) route, or hydrogen atom transfer (HAT) [10]. A laccase—HBT (1-hydroxybenzothiazole) system was used in depitching processes in the paper industry [11]. Nevertheless, the toxic compounds generated and the cost of the artificial mediators have led to its disuse [12]. Additionally, synthetic mediators are well known for their side effects. They have an inactivating influence on enzymes, due to interactions with the proteins [13]. As a consequence, side-products disable the mediator structure, producing a non-functional substrate.

A new tendency in the use of mediators is to take advantage of the products derived from the biodegradation process, when fungal laccases are involved. These compounds contribute to the total lignin reaction. Some examples are the syringaldehyde, p-hydroxycinnamic acid and vanillin [14]. The phenoxyl radicals generated from these structures can oxidize non-phenolic residues of lignin, destabilizing the polymeric network. 

LMS delignification technologies have found practical applications, such as the lignozym process to pulp biobleaching [15]. LMS systems have the ability to promote biografting reactions. Leonowicz reported that laccases possess both a polymerizing as well as a depolymerizing activity, depending on the size of the substrate in terms of its molecular weight [16]. This suitability to polymerize lignin has gained interest in binding reactions [17] for enzymatic adhesion of fibers and the manufacture of composite materials.

Grafting of phenols of different kinds of fibers may enhance physicochemical properties of different reported materials, such as strength. This is by new hydrogen bonding and cross-linking between phenoxyl groups on the surface [18]. In catechol-polymer hydrogels containing Fe^3+^, the covalent and coordination bonds formed are regulated by pH during the constitution of the catechol-polymer hydrogel [19,20]. At pH greater than 5, Fe^3+^–catechol coordination bonds mechanically enhanced the covalent network. In another study, through anionic polymerization, a strategy was designed to control the functionalization of polyethylene oxide (PEO)-based polymers with catechol [21]. Tannic acid (TA) was used as a molecular glue in the preparation of DNA hydrogels due to the presence of ester bonds and ten pyrogallol moieties [22]. A tunicate-mimetic hydrogel adhesive based on a chitin nanofiber/gallic acid composite was reported. The pyrogallol group-mediated cross-linking, along with nanofibrous structures, improved the dissolution resistance and cohesion strength of the hydrogel in wet conditions [23]. Copolymers synthesized bearing gallol groups exhibited stronger adhesive performance than the catechol-functionalized copolymers under all tested conditions. The higher binding strength suggested superior tridentate-related interfacial interaction and chemical cross-linking [24] Holten-Andersen and his group have described a strategy for introducing mono-, bis- and/or tris-catechol–iron^3+^ cross-links into a synthetic polymer network (PEG-dopa4) and demonstrated that it displayed high elastic moduli [19]. The stoichiometry of the complexes was controlled by pH. The deprotonation of catechol hydroxyls determined the catechol–iron ratio of the complex. For comparison, a covalently cross-linked polymer gel was prepared using NaIO_4_-induced oxidation of the polymer. The authors found the iron–catechol cross-linked gel reestablished its stiffness and cohesiveness within minutes after failure, just as has been observed in other types of reversible polymer networks. The covalent union attained by the NaIO_4_-induced oxidation was evidenced by an EDTA treatment, where tris-catechol–iron cross-linked gel and a covalent gel were exposed to a concentrated solution of EDTA, and only the former was dissolved.

The tris-catechol–iron gel has a self-healing behavior, where the reversibility of the union allows reconstitution of the material when the pH is changed. When chemical oxidation is performed, the covalent bond occurs, and thus the self-healing capacity is lost. Enzyme-catalyzed systems offer ubiquitous advantage for the redox process where no additional chemical sub-products are generated. When using periodate for the oxidation, iodine is produced during the reaction. Laccases proceed by the oxidation of the substrate to the reactive radical. The overall outcome of the catalytic cycle is the reduction of one molecule of oxygen to two molecules of water, and the concomitant oxidation of substrate molecules to produce radicals. These reactive molecules produce the oligomers and polymers. In this regard, enzyme cycles are a green alternative for oxidation reactions. Felby has demonstrated the ability of laccase to improve the properties of fiber board, creating phenoxy radicals at the fiber [25]. Recent findings have been reported regarding the heterogeneous reaction of phenolic compounds in the presence of laccase with high-yield kraft pulp. Hence, laccases have been evaluated in several grafting reactions, allowing bonding of phenolic compounds adding special properties to the paper, such as increasing its strength. For example, the reaction with 4-hydroxybenzoic acid increased the surface carboxylic acid groups and modified the molecular weight of the material [26,27,28]. Moreover, they attained an augmentation in burst- and tensile strength. Lund and coworkers have shown how delignification reactions produce an increase in wet tensile strength. They suggested this was caused by radicals produced by the laccase–mediator combination, which promote covalent binding of the mediator and the lignocellulosic matrix. A patent [29] about the coupling reaction among many biopolymers was reported in the presence of peroxidases. They described an enzymatic treatment to cellulosic fibers adding a phenolic carboxylic acid to increase the negative charge of the material, and an ionically charged strengthening agent such as carboxymethyl cellulose (CMC). 

This work explores the improvement of polymerization and mechanical properties of paper by introduction of a series of low-molecular-weight mediators such as gallic acid, vanillic acid and catechol in a coupled insertion reaction where both carboxymethyl cellulose and the lignocellulosic fiber are bio-grafted using a commercial laccase. The presence of chitosan was assessed for a constant composition of CMC, fiber and laccase. For the adequate selection of a mediator, several parameters were considered. Solution chemistry and redox potential of the compounds involved in the reactions were key factors for generating the reactive species to promote biografting. Our study intends to fill the gap regarding the use of non-synthetic mediators, developing a friendlier process to strengthen paper fibers and understand the polymerization reactions that affect paper characteristics. 

## 2. Materials and Methods

### 2.1. Lacasse Biografting

Cellulose pulp was taken directly from the paper machine located at Pachisa-Copamex, Chihuahua, Mexico (28°39′25.9″ N 106°03′49.4″ W). Raw material was provided from the production of Kraft paper. The recycled material, mainly cellulose of second generation, was pulped and treated before it was introduced to the paper machine. Pulp was treated previously to the paper formation and frozen until it was processed. Pulp was washed with acidified water for 30 min and deionized water at 1% consistency prior to the enzymatic treatment, and following filtration and reconstitution, pH was adjusted manually adding either HCl or NaOH (pH = 6.3 ± 0.2). Laccase treatments (MAXIMYZE 2054, Buckman^®^ [30], Memphis, TN, USA) were performed in an aerated 2 L reactor (Pyrex) manufactured by Copamex (Chihuahua, Chihuahua, México). A total amount of 24 kg of enzyme per fiber ton was used, as recommended by the supplier. Reactants were added to 1 L of the pulp (consistency 1%: 10 g L^−1^) at 80 °C in the following order: 0.2 mL of enzyme for 105 min, 2 mmol of mediator (Sigma-Aldrich Corp, St. Louis, MO, USA), and when it proceeded, 2.7 mL of chitosan (1.85% *w*/*v*) for 10 min, and afterwards, 1.4 mL of carboxymethyl cellulose (Sigma-Aldrich Corp, St. Louis, MO, USA) (4% *w*/*v*) for 10 min. Redox potential and pH was monitored for each step of the process, and the latter was kept under 6.5 at the beginning of the reaction. Once the reaction finished, the pulp was poured into a mesh and the water was withdrawn by vacuum, pressed for 7 min and dried at 100 °C for 8 min. Hand sheets were prepared in a former (Rapid-Köthen Tendring Pacific, Saffron Walden, UK) according to ISO 5269-2.

All treatments were also made in the absence of mediator to have the respective controls. Mediators evaluated were gallic acid (GA), vanillic acid (VA) and catechol (1,2–DHB) (all provided by Sigma-Aldrich Corp, St. Louis, MO, USA); at two levels of chitosan (0% and 0.005%). For instance, a total of 6 treatments and their replicates were performed. 

### 2.2. Paper Properties Evaluation

#### 2.2.1. Mechanical Tests

Potential flat crush resistance according to CMT test Tappi 809 om-99 took place for assessing crush resistance before paper manufacturing. Ring crush test Tappi 822 om-02 was used for correlating edgewise compression strength of paper. Paper bursting strength was analyzed by Mullen test Tappi 403 om-97 inside of a 50–1200 Kpa range and in the form of 0.6 mm-thick flat sheets. Tensile paper and paper properties (tensile strength, stretch, tensile energy absorption and tensile stiffness) considering a constant rate of elongation were evaluated at a tension test Tappi 494 om-01.

#### 2.2.2. Scanning Electron Microscopy (SEM)–Energy Dispersive Spectrometry (EDS)

A SEM (SEM JSM-5800LV, JEOL, Tokyo, Japan) was used and operated at 15 kV, 0° incidence. A sputtering (Denton vacuum Desk IV Gatan, Pleasanton, CA, USA) was used to cover with gold the paper analyzed. SEM electron-beam scans the surface of the sample.

#### 2.2.3. Confocal Laser Scanning Microscopy (CLSM)

Images of paper treated and their controls were taken with an LSM 700 confocal microscope (Inverted Zeiss LSM 700, Carl Zeiss, Jena, Germany) using the 10× and 20× objectives. Paper was dyed with acid fuchsin (1.0%) previous to the analysis.

#### 2.2.4. Fourier-Transformed Infrared Spectroscopy (FTIR)

An attenuated total reflectance (FTIR/ATR, DuraSamplIR II, Spectrum GX/Smiths, Perkin Elmer, Waltham, MA, USA) spectrometer was used; 32 interferograms were co-added to get spectra, scanning from 550 to 4000 cm^−1^ at 4 cm^−1^ resolution. Analysis of controls and treated papers was performed for comparison.

#### 2.2.5. Nuclear Magnetic Resonance (^13^C NMR)

All the experiments were performed with a Brucker Ascend TM 400 NMR (Brucker, Bellerica, MA, USA) magnetic field of 7.05 Teslas at 400 MHz, tubes equipped with an AVANCE III NanoBay software (topspin pI7) and using 5 mm outer sample tube (O.D.). The analysis temperature was 45 °C, and spectra were acquired with 12,000 accumulations and acquisition time of 12 h. Raw and treated paper samples (30 mg) were dissolved in 1 g of ionic liquid 1-Ethyl-3-Methylimidazolium Bromide (obtained in the laboratory synthetically) in the presence of deuterated H_2_O. The ^13^C NMR spectrum was recorded. 

## 3. Results

### 3.1. Lacasse Biografting and Mechanical Properties

**Scheme 1:**
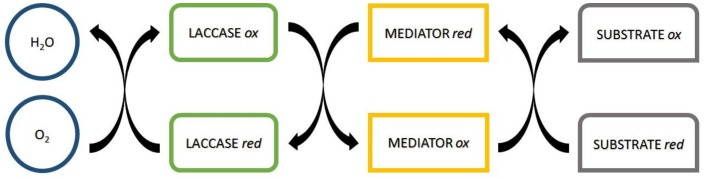
Laccase activity and the role of the mediator and substrate.

Conventionally, laccases were used for delignification in the bleaching process of paper [31,32]. As they can depolymerize the lignin structure, they also can undergo coupling and insertion reactions [12,33]. The mechanism is similar to the one involved in lignin synthesis. Laccases perform a one-electron oxidation of the substrate. Once oxidized (laccase ox), it interacts with the phenolic substrate (mediator or any other substrate with lower chemical potential) to generate the oxidized species (Scheme 1). 

In order to have the oxidation, the laccase’s redox potential has to be higher than the substrate’s redox potential [34]. The reported redox potential of laccases ranges from 0.662 to 1.030 V (E° Ag/AgCl) [35,36]. The more-positive the electrode potential is, the greater the tendency of the oxidized form to be reduced. As an oxidant, laccase activity is enhanced by the presence of electron-donating substituents in the substrate. We selected gallic acid (GA) as a tri-hydroxyl system with an acid functionality; vanillic acid (VA) with two hydroxyl moieties and an acid functionality; and finally, catechol (1,2–DHB) showing two hydroxyl groups bonded to the aromatic structure. The documented potentials (E° Ag/AgCl) for these mediators are 0.550 V for GA, 0.901 V for VA and 0.501 V for 1,2–DHB [37]. The potential of the experimental system depended upon the components in solution, and thus, the variation of redox potential (ΔE) was assessed by measuring the potential at the beginning and at the end of the paper treatment (Table 1). 

The CPX (Chitosan) system showed lower potentials in all cases, caused by the reducing environment. This potential diminished in the following order: GA > VA > 1,2–DHB. This might be caused by the final compounds influencing it: the enzyme, soluble lignin, the compounds added to the mixture that did not react with the fiber (CMC and CPX), and the reduced species.

The production of compounds with electron-donating capacities was favored when CPX was present; that is, the redox reaction between the enzyme and the biopolymers proceeded to a greater extent. When laccase was oxidized, oxygen was converted to water, and the resulting reduced enzyme underwent oxidation of the mediator (Scheme 1). When adding chitosan and carboxymethyl cellulose to the mixture, additional grafting was reached among the hydroxyl radicals and the amino groups.

The interaction was confirmed through the mechanical properties measured. Results of the CMT, ring crush, Mullen and tensile strength tests are described in Table 1. In all treatments, an improvement of the mechanical properties was attained. For the case of GA, the four parameters improved more than 40% compared to the blank hand sheets. Moreover, ring crush improved more than 120%.

When using VA, the mechanical properties were enhanced from 74% to 88% when CPX was added, while treatments without CPX improved from 10% to 83%.

Mechanical properties were improved with 1,2–DHB, but no significant difference was observed when CPX was used.

Lund and Felby found that the treatment with laccase did not have any effect on the wet strength of the pulp, whereas addition of lignin increased it after enzymatic treatment [38]. The strength is generated as a product of the network established by all the biopolymers grafted and cross-linked around the fiber. They suggested that a mediated oxidation process releases lignin from the fiber wall, the former of which polymerizes and precipitates on the fiber surface. They also reported that the wet strength is dependent on the time of contact between pulp and the enzyme/mediator system. Dry tensile strength in paper is originated by the hydrogen bonding among fibers. 

The laccase treatments made by Aracri and coworkers [2] increased wet tensile strength 15% and dry tensile strength 5.5%. They also suggested a potential formation of water-resistant covalent bonds between fibers by coupling of phenoxy radicals in the sheets. 

**Scheme 2:**
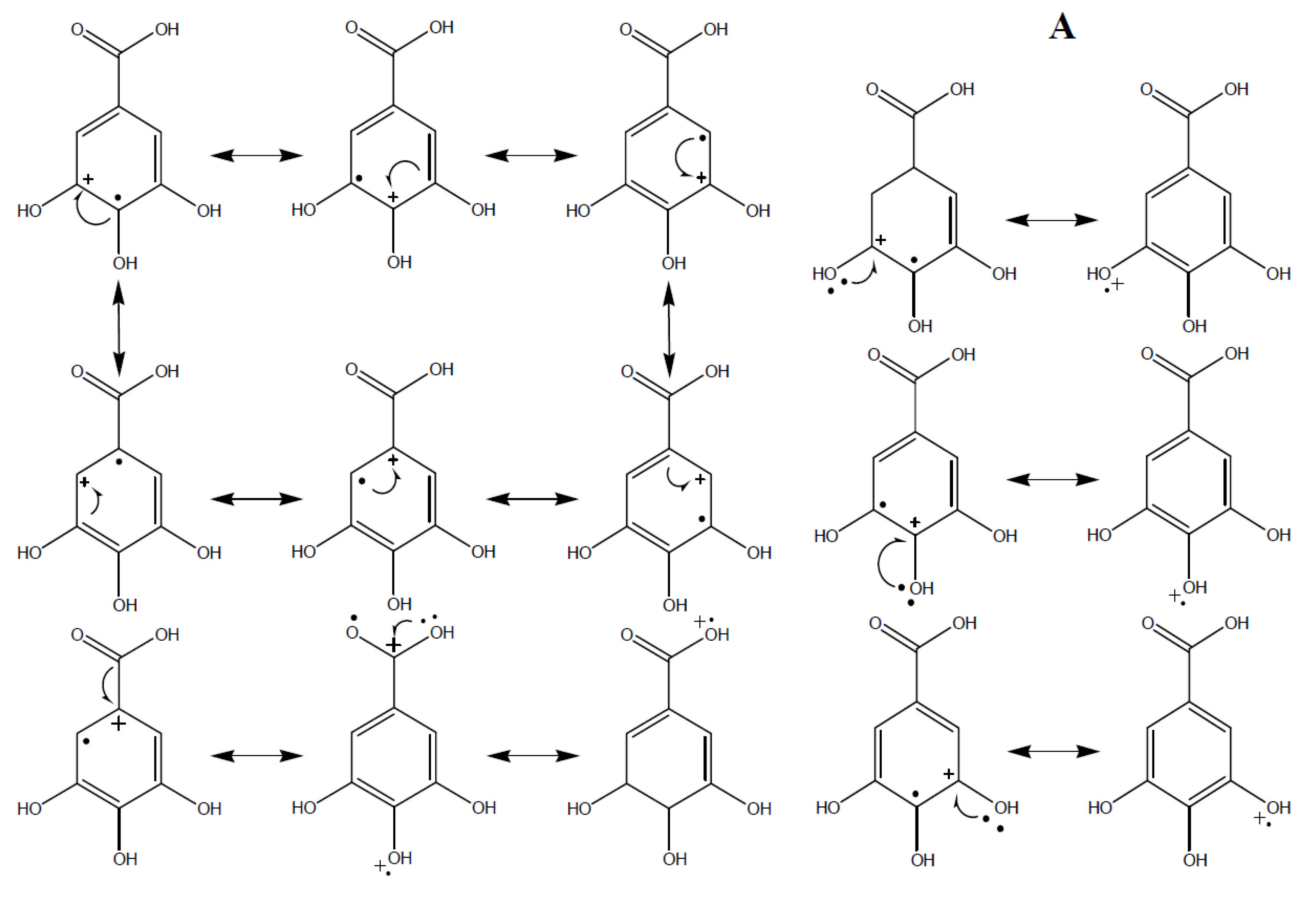

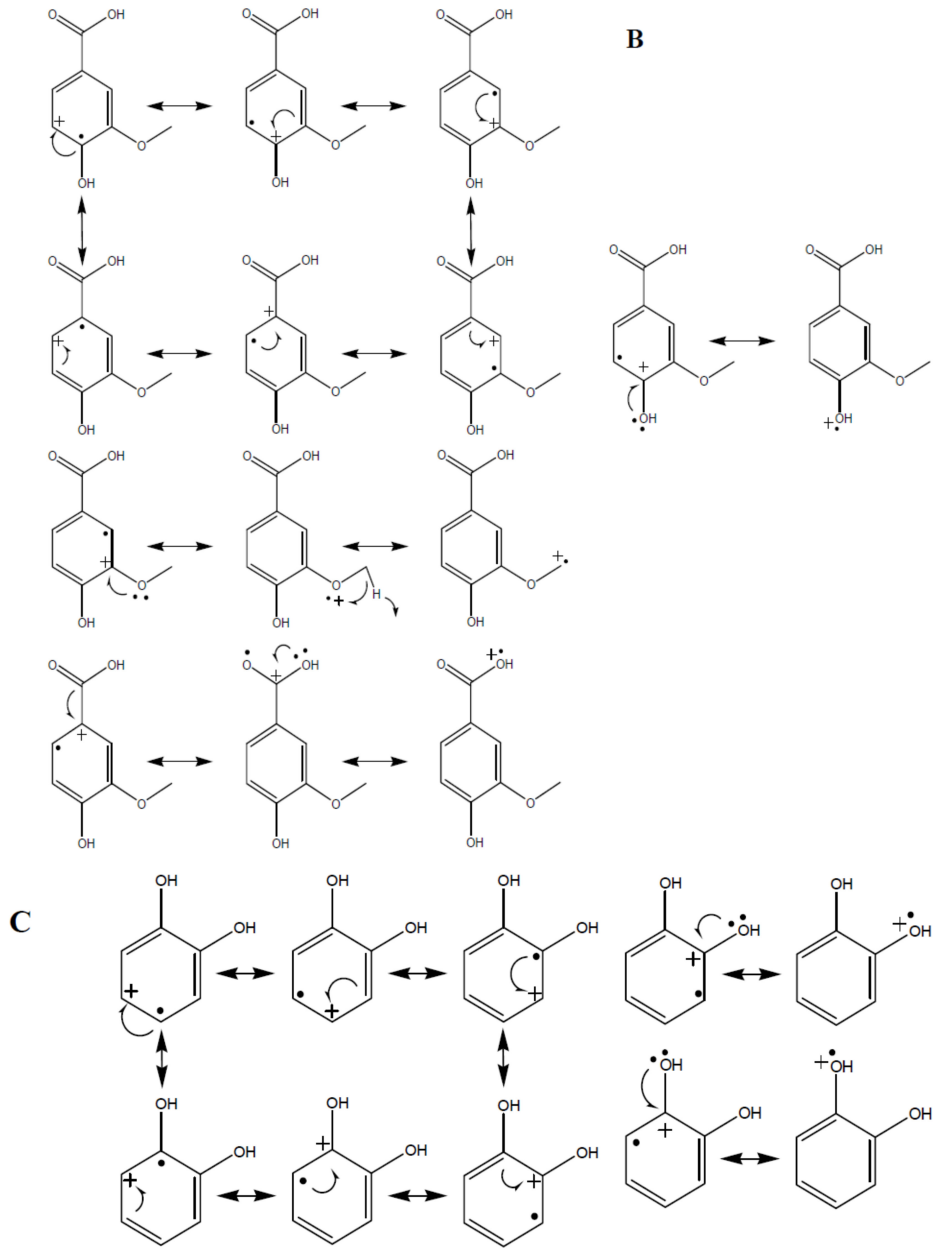
(**A**) Resonance structures of gallic acid radical; (**B**) resonance structures of vanillic acid radical; (**C**) resonance structures of catechol.

One of the reported routes for the oxidation of the mediator is through an electron-transfer mechanism [10]. Laccase withdraws one electron from the mediator, generating a cationic radical which stabilizes through the resonance structures. The structures and radicals formed after subtraction of one electron of the mediators are presented in Scheme 2A–C. Gallic acid presents fifteen resonance structures, meanwhile vanillic acid, fourteen, and finally 1,2–DHB, ten structures. Resonance structures may have an influence on the grafting reaction through the stabilization of the reactive compound. This can be explained since the mediator acts after being oxidized (med ox) by the enzyme and further oxidizes any substrate with a lower chemical potential. Med *ox* could initiate a radical coupling polymerization or a nucleophilic addition [39]. This could explain why GA is better in performance than VA and 1,2–DHB.

**Scheme 3:**
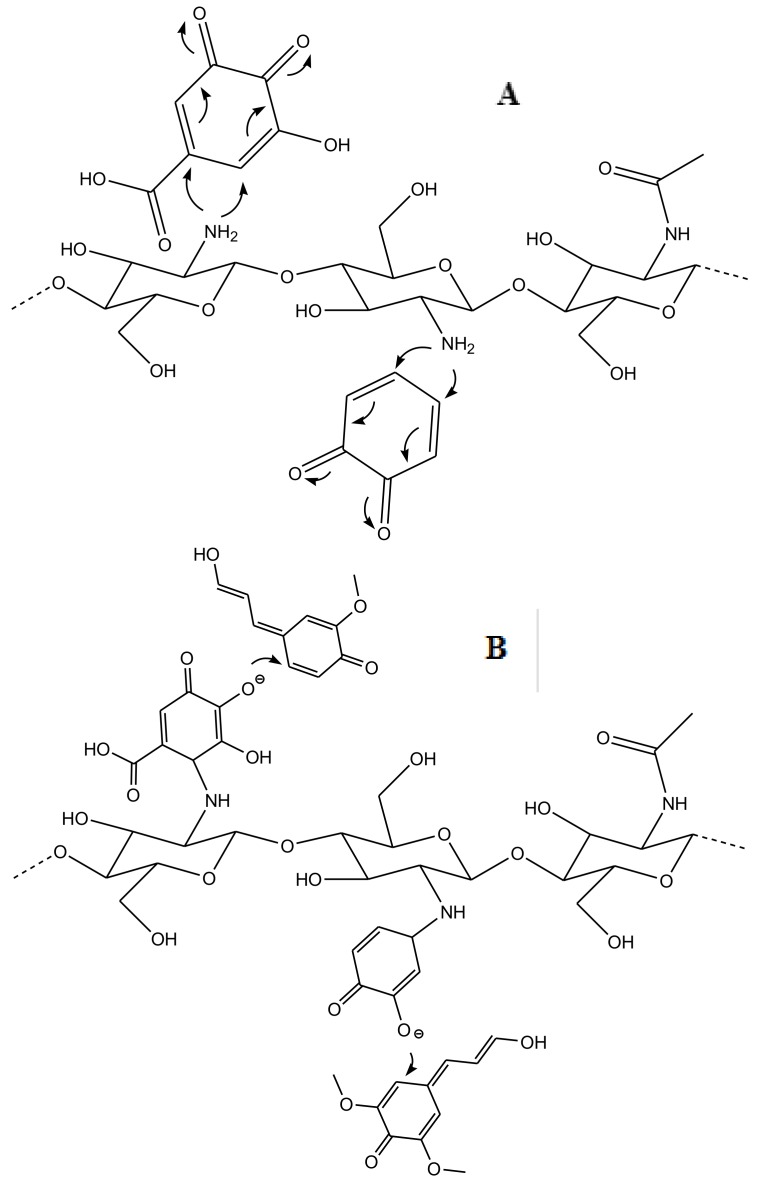
(**A**) Interaction of the mediator’s quinones with chitosan amine groups; (**B**) lignin’s quinones interact with mediator’s quinones.

The proposed mechanism is depicted in Scheme 3A,B. Med *ox* further reacted with chitosan (CPX). Chitosan is a derivative of chitin, after deacetylation under alkaline conditions. The process leads to a random distribution of acetylated/non-acetylated units along chitosan chains. Chitosan consists of pyranose cycles of β-(1,4)-2-amino-deoxy-β-d-glucose. The (M–O) radicals obtained from the mediators are finally stabilized into electrophilic *o*-quinones, which could undergo reaction with the amino groups of chitosan (nucleophilic character), forming an amide covalent bond.

**Scheme 4:**
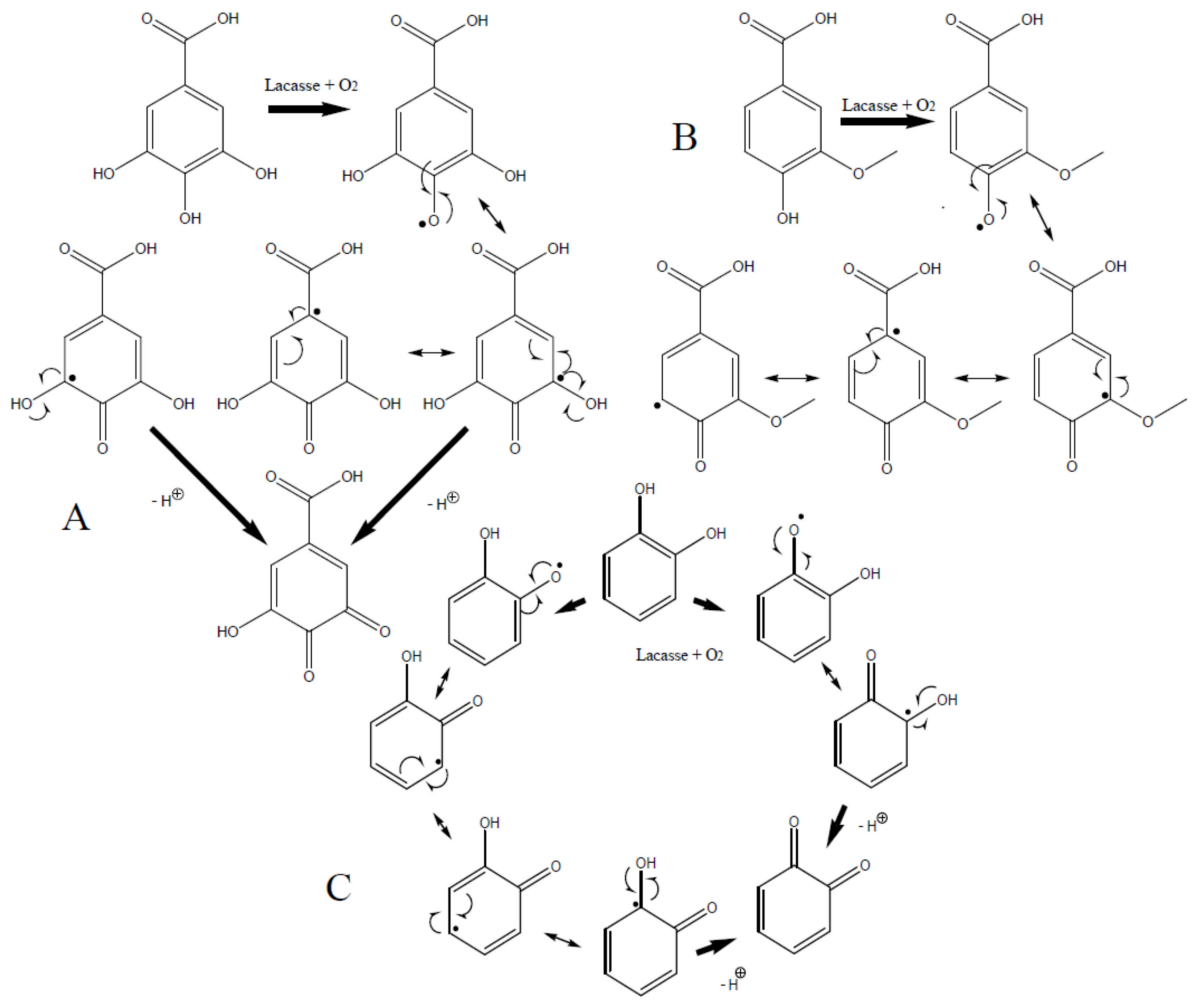
HAT (Hydrogen Atom Transfer) mechanism and quinone formation. (**A**) gallic acid; (**B**) vanillic acid; (**C**) catechol.

Another reported mechanism is the radical H abstraction route of oxidation (HAT), where the initial step is the conversion of the mediator into a radical cation by mono-electronic enzymatic oxidation. Deprotonation of the radical cation of the mediator then follows, giving the corresponding (M–O·) radical. The latter abstracts the benzylic hydrogen from the substrate, giving rise to the oxidized molecule (Scheme 4). Polymerization is thus promoted among the reactive quinones generated.

After the mechanical test, a characterization was made of the morphology, to visualize the changes of the fibers and the physicochemical interaction among the biopolymers that we are suggesting. Results are presented in the following sections.

### 3.2. Paper Characterization

The principal objective of thermal analysis was to identify the displacements in the degradation profile and the fixed carbon preserved. In the first case, the crystallinity alterations of cellulose were evidenced. In the second one, an increase of the relative mass of the lignin due to the biografting was noticed. Table 1 shows the differences in the thermograms. As can be seen, samples with CPX presented more fixed carbon than the ones without CPX. This might be caused by an increase in the lignin mass, which is a thermally stable compound.

**Figure 1:**
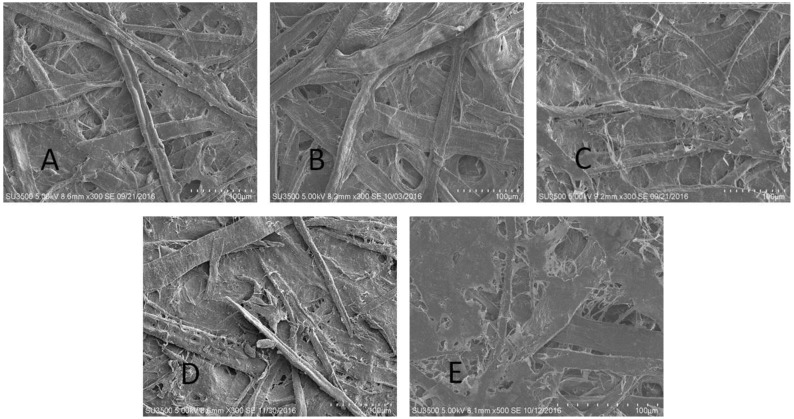
(**A**) SEM (Scanning Electron Microscope)) of the blank handsheet, 300× magnification; (**B**) SEM of the sample vanillic acid, without CPX (Chitosan), 300× magnification; (**C**) SEM of the sample 1,2–DHB (Catechol) without CPX, 300× magnification; (**D**) SEM of the sample gallic acid without CPX, 300× magnification; (**E**) SEM of the sample gallic acid with CPX, 300× magnification.

Morphology was assessed by two different microscopies, that is, SEM and confocal microscopy. The blank and all the papers were first observed by SEM. Figure 1A–C displays the hand sheets obtained without mediator and using VA and 1,2–DHB. In Figure 1D,E, the micrographs for GA with and without CPX are shown.

**Figure 2:**
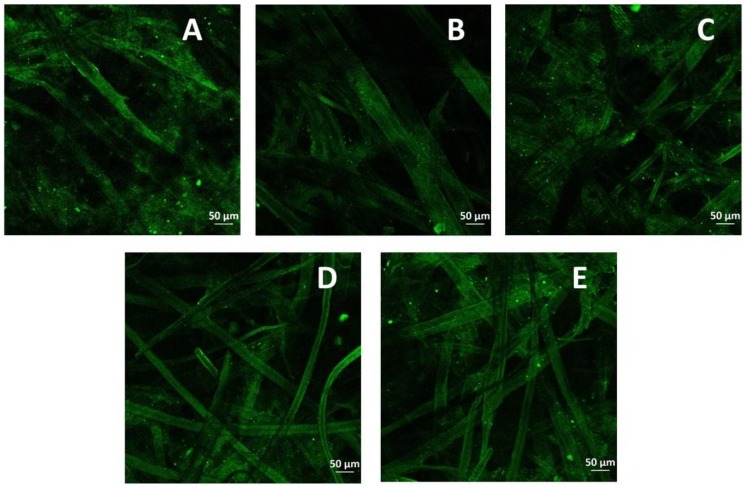
(**A**) SCEM (Scanning Confocal Electron Microscopy) blank; (**B**) SCEM for 1,2–DHB; (**C**) SCEM for vanillic acid; (**D**) SCEM for gallic acid–CPX; (**E**) SCEM for gallic acid–CPX. Scale indicates 50 microns.

Confocal microscopy uses a laser capable of penetrating into the material under study. The micrographs of the six hand sheets, comparing the treatment with three mediators with and without CPX, are displayed in Figure 2. It is important to emphasize that there were no color changes among the papers macroscopically (Figure S1).

The longer fibers measured are those obtained using gallic acid and CPX. In these materials, lignin appeared precipitated along the structure. Finely dispersed dots were visualized with an average diameter of 10 nm. This issue is in accordance with reported data [2], where lignin is depolymerized from the biomass during laccase treatment, and liberated to the solution.

Once lignin was free from the other biopolymers in the fiber, it underwent a polymerization reaction promoted by the enzyme, and re-precipitated on the fiber structure. The same observation was made analyzing both micrograph techniques. The GA and CPX hand sheets presented less-loosely attached fibers in the paper. Hence, CPX seemed to have a gluing effect over the paper surface. Traditionally, wet strength is increased by adding resins such as PAE (polyaminopolyamide–epichlorihydrin). This additive adds a fixed retention capacity into the fibers. Exceeding the limits imply PAE drainage from paper, and the excess passes into the white water of the papermaking system and causes operational, economic and environmental problems. For the material, development of a gluing effect is evident. The structure does not allow any possibility of leaching.

**Figure 3:**
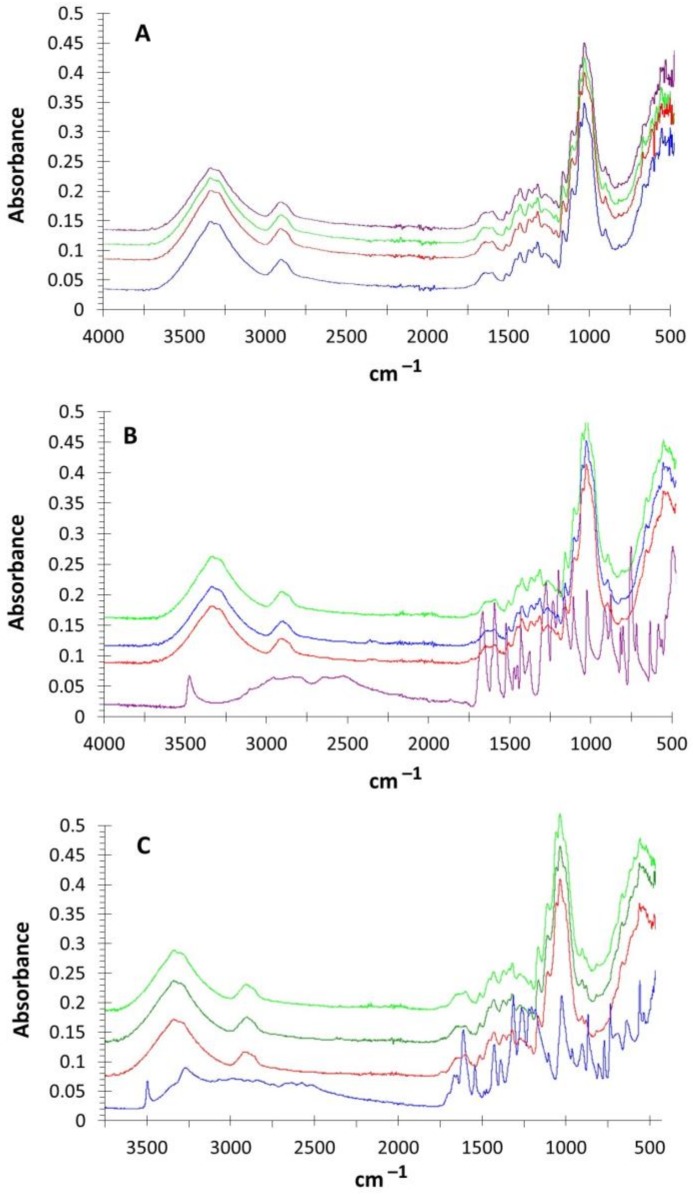
(**A**) Infrared spectra for blank (purple), catechol mediator (green), paper treated with laccase, 1,2–DHB and CMC (Carboxymethyl cellulose) (red); (**B**) infrared spectra for blank (green), vanillic acid mediator (purple) and sample of VA (Vanillic acid) and CPX by duplicate in blue and red; (**C**) infrared spectra for blank (green), gallic acid mediator (blue) and sample of GA by duplicate in dark green and red.

In Figure 3A, the results of the FTIR analysis are shown for the paper treated with 1,2–DHB. There was no variation among the blank, control and treatment with 1,2–DHB. Vila and coworkers [40] claimed that characteristic bands at 897 and 1273 cm^−1^ could be indicative of amorphous cellulose. On the other hand, bands at 1370 and 1430 cm^−1^ correspond to crystalline cellulose, and they were equally distinguished in the four samples. However, other bands of the crystalline cellulose phase were not observed in any case (700, 750 and 3267 cm^−1^). The presence of lignin in the pulp was evidenced by bands at 1460, 1510 and 1600 cm^−1^, with no variation among the blank, control and treatments. Finally, two bands corresponding to vibrations of the alcohol group were presented at 3290 and 3333 cm^−1^. The latter is reported at 3400 cm^−1^ and its shift to lower values indicates the difference in the hydroxyl’s substitution. 

In Figure 3B,C, the spectra for papers treated with vanillic and gallic acids are shown. Bands specified in Table 2 are found for all the papers tested. There was no increase in the aromatic, ether and carboxyl signals (C–O and C=O) for all the samples modified by the VA system and GA system. No increase in the methoxyl bands was observed. The presence of the lignin bands (aromatic ring) showed that there was no depolymerization of the lignin by the laccase. In all cases, chitosan signals were observed, especially in the bands that follow: 1590 cm^−1^ assigned to N–H bend in secondary amine; 1654 cm^−1^ assigned to N–H bend in primary amine; 3460 cm^−1^ assigned to N–H constriction.

**Figure 4:**
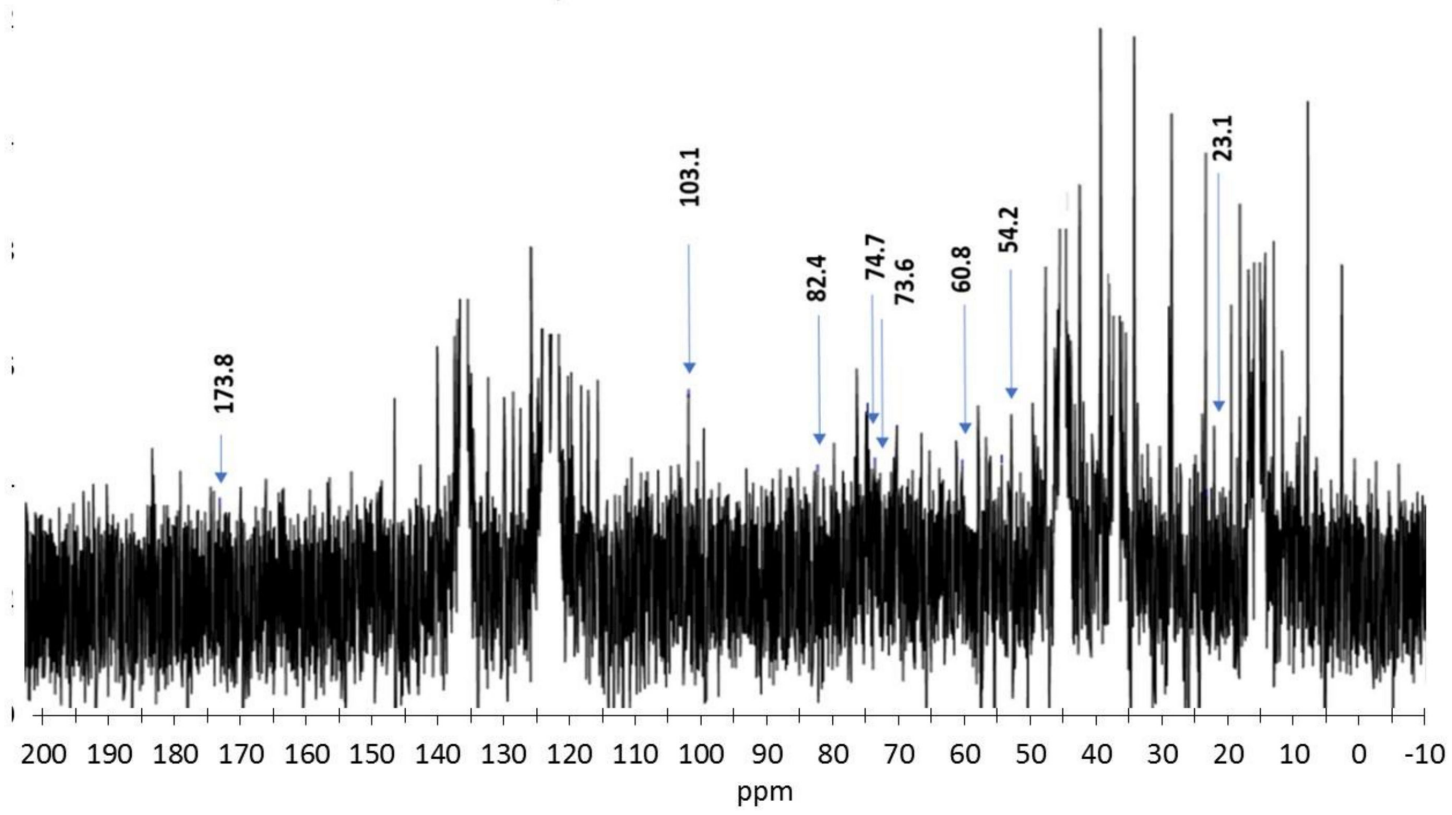
^13^C NMR spectrum. Chitosan chemical shifts stand out.

The ^13^C-nuclear magnetic resonance spectrum is displayed in Figure 4. Detailed analysis of carbon-specific functional-group signals are presented in Table 3.

As can be observed, some chemical shifts can be assigned to the chitosan polymer, revealing the N–C–O (C8) at 173.82 ppm, C2, C3, C5, C6, chitosan glucosidic bonds, and N–CH_3_ (C7) at 23.12 ppm. Lignin presented some chemical displacements very different from those corresponding to chitosan; all thess in the interval of 100 to 175 ppm, which is associated with lignin phenolic subunits. The peak at 152 ppm may be attributed to the carbon of the C–OH or C–O–CH_3_ groups in the syringyl units. As a result, the integration of the chitosan into the paper structure was then evident. The C2 in the chitosan glucosidic ring bonded to amine corresponds to the structure depicted in Scheme 3.

Few reports on composite study by ^13^C NMR are found in the literature. Crouvisier-Urion [41] and coworkers reported the analysis of a chitosan–lignin film composite generated by solvent evaporation. Molecular interactions of chitosan–lignin were investigated by ^1^H–^13^C cross-polar solid-state NMR. Although the lignin signals do not appear to interfere with those of chitosan, the lignin contribution spectrum evidenced dipole–dipole proton–carbon interaction. In particular, a variation in the relative intensity of the displacement was observed for the three carbons involved in lignin O–CH_3_ (56 ppm), Cα lignin (72 ppm) and C=O chitosan (175 ppm). The latter modification at the carbonyl correlated well with the previous rearrangement of the polymer on the surface, as revealed by XPS (X-Ray Photoelectron Spectroscopy) analysis. Although there were no strong interactions between lignin and chitosan, a few dipole–dipole interactions were not excluded, particularly those involving the carbonyl of chitosan and the Cα-hydroxyl of lignin. These interactions could weaken the chitosan network, which in turn led to the reduction of mechanical properties.

In other work, a hybrid material was obtained by the reaction between chitin and lignin in a 1:1 mass ratio [44]. The ^13^C-CP–MAS–NMR (Cross Polarization—Magnetic Angle Spinning—Nuclear Magnetic Resonance) spectrum of chitin before modification exhibits the intense and narrow signals characteristic of α-chitin, while lignin showed wide but less-intense signals. The modification of chitin with lignin was a characteristic feature in the spectrum. Slight chemical shifts were also observed, confirming the hypothesis that chitin and lignin are connected via hydrogen bonds. Hydrogen bridges were possible because of the significant functional groups present primarily in the Kraft lignin.

## 4. Conclusions

Laccase biografting was attained by using a commercial enzyme along with the following components: a mediator (gallic acid (GA), vanillic acid (VA) or catechol (1,2–DHB)); carboxymethyl cellulose; chitosan; and Kraft pulp fiber. An improvement in the mechanical parameters was obtained depending on the biopolymers coupled and mediator selected. A correlation between the mediator effectiveness and electrochemical potential was found. Lower final potential promoted better performance, particularly in the systems with CPX, which in all cases produced a reducing environment. ΔE was observed in the following order: GA > VA > 1,2–DHB. This fact was explained in terms of the final compounds in solution. GA presented more stability than the other polyphenols due to the resonance structures of the (M-O·) radical, which could produce radical coupling polymerization or a nucleophilic addition. The larger number of resonance structures, the better their performance. In all treatments, an improvement of the mechanical properties was attained. In the case of GA, ring crush improved more than 120%. In the case of VA, mechanical properties were enhanced from 74% to 88% when CPX was added. Some coupling reactions were suggested, and the final polymer structure presented a covalent bonding between CPX and the highly reactive quinones. Thermal analysis evidenced more fixed carbon, which was related to lignin. This compound did not alter its main structure, and FTIR analysis showed the main signals for a well-polymerized lignin structure. By microscopy, longer fibers were visualized for CPX and GA paper. Lignin dots also were shown by SEM. ^13^C-nuclear magnetic resonance spectra evidenced some chemical shifts, which could be assigned to the chitosan polymer. The integration of the chitosan into the paper structure was evidenced. The C2 in the chitosan glucosidic ring, bonded to amine, corresponds to the structure suggested. This is the first study where biografting produced covalent bonds between fibers by coupling of phenoxy radicals in the sheets, using chitosan, CMC and Kraft cellulose fibers for strengthening paper.

## Figures and Tables

**Table 1:** Redox potential and mechanical properties for the papers evaluated.

Mediator	CPX * × 10^3^	CMT * lb/6 Pulg	Ring crush lb/6 Pulg	Mullen lb/6 Pulg^2^	Tension kg/15 mm	ΔE (mV)	Thermal Profile
Gallic acid	0	50.03	35.14	59.93	11.10	42.2	−5% HA *
Gallic acid	5	52.53	35.87	92.45	8.48	−44.4	+4% CF *
Vanillic acid	0	49.01	27.44	75.10	9.33	66	+4% CF
Vanillic acid	5	57.08	43.07	77.16	10.17	4.4	
Catechol	0	46.38	35.93	65.83	11.24	41.6	No change
Catechol	5	64.88	34.13	71.47	8.74	−21.3	
Blank	0	41.0	24.8	41	5.70	NA *	Reference

* CPX, Chitosan; CMT, Flat crush of corrugating medium test; HA, humidity; CF, fixed carbon; NA, not applicable.

**Table 2:** FTIR assignments for the spectra acquired.

Functional Group	Vila, 2011 [40]	Functional Group	Thakur, 2015 [42]	Functional Group	Thakur, 2016 [43]
Amorphous cellulose	897 cm^−1^	Vibration C=O in different spatial direction	944 cm^−1^	Stretching C–H out of plane	852 cm^−1^
Amorphous cellulose	1273 cm^−1^	Stretching C–O by grafting	1244 cm^−1^	Asymmetric stretch ether C–O–C	1034 cm^−1^
Crystalline cellulose	1370 cm^−1^	C=O vibration in different spatial direction	1327 cm^−1^	Carbonyl stretch C=O	1276 cm^−1^
Crystalline cellulose	1430 cm^−1^	C=O vibration in different spatial direction	1449 cm^−1^	Methoxy group C–O–CH_3_	1379 cm^−1^
Lignin	1460 cm^−1^	Aromatic skeletal vibrations	1449 cm^−1^	Methoxy group C–O–CH_3_	1466 cm^−1^
Lignin	1510cm^−1^	Aromatic skeletal vibrations	1512 cm^−1^	Aromatic stretching C–C bond	1516 cm^−1^
Lignin	1600 cm^−1^	Double substitution p-aromatic ring	1602 cm^−1^	Carbonyl stretching C–C bond	1600 cm^−1^
Stretching C–H	2900 cm^−1^	Double substitution p-aromatic ring	1628 cm^−1^	C=O by phenolic insertion	1665 cm^−1^
Face, Iα	3267 cm^−1^	Carbonyl group C=O	1673 cm^−1^	C=O by phenolic insertion	1691 cm^−1^
Vibrations O–H	3290 cm^−1^	Stretching C–H	2924 cm^−1^	Carboxyl group	2500–3000cm^−1^
Vibrations O–H	3333 cm^−1^	Vibrations O–H	3380 cm^−1^		

**Table 3:** ^13^C NMR assignments from the spectra and reported data for chitosan.

Signal no.	Sample ppm	Lignin Characterization Reference, ppm	Functional Group Assignment
1	173.82	173.6–173.8	N–C–O (C8) in chitosan
2	146.99	146.8	C4 in non-esterified lignin guayaquil
3	140.49	140.8–140.4	Structure lignin A(4), B(1) and D(1)
4	137.89	138.2	C1 from esterified lignin syrigil
5	132.76	132.4–132.6	C5 in ether bond 5-5
6	128.98	129.3	Cβ in lignin aryl group–CH=CH–CHO
7	127.89	128.0	Cα y Cβ in lignin aryl group–CH=CH–CH_2_OH
8	126.24	125.9	C5 in non-esterified lignin bond 5-5 no
9	120.68	120.5–120.7	A6, B6 from lignin
10	120.14	119.4	C6 in a guayaquil lignin unit
11	118.65	118.4	C6 in a guayaquil lignin unit
12	116.11	116.5	B5 from lignin
13	103.14	102.7–105.7	C1 chitosan glucosidic bond
14	82.46	80.9–85.7	C4 in chitosan glucosidic ring
15	76.79	76.5–77.1	Cα in β-1 (1,2 disyringilpropane-1,3-diol acetate) from lignin
16	75.37	75.5	Cα in guayaquil-*O*-aryl from lignin
17	74.73	74.7	C5 chitosan glucosidic bond
18	73.61	73.1–75.7	C3 chitosan glucosidic bond
19	60.84	59.6–60.8	C6 chitosan glucosidic bond
20	54.23	55.2–57.6	C2 in chitosan glucosidic ring bonded to amine/amide
21	39.74	41.0–41.2	Cα in β–β lignin open bond
22	29.38	29.2	CH_2_ in lignin aliphatic lateral chain
23	23.12	22.8–23.3	N–CH_3_ (C7) in chitosan
24	19.88	20.5	CH_3_ acetoxy from lignin
25	13.43	14.0	γCH_3_ *n*-propyl from lignin side chain

## Supplementary Materials

The following are available online at http://www.mdpi.com/2073-4360/9/11/570/s1, Figure S1. (A) Paper with no treatment. (left); (B) Paper treated with Lacasse, Gallic acid, CPX, and CMC (right).
