# Supplementary material for: Effect of Laccase-Mediated Biopolymer Grafting on Kraft Pulp Fibers for Enhancing Paper’s Mechanical Properties
Source: Polymers (Basel). 2017 Nov 2;9(11):570. doi: 10.3390/polym9110570 (PMC6418782; doi:10.3390/polym9110570)
Supplement: Supplementary file 1 [file polymers-09-00570-s001.pdf]

Supplementary Materials: Effect of Laccase-Mediated Biopolymer Grafting on Kraft Pulp Fibers for Enhancing Paper's Mechanical Properties

Lourdes Ballinas-Casarrubias, Luis Villanueva-Solí, Carlos Espinoza-Hicks, Alejandro Camacho-Dávila, Hilda Amelia Piñón Castillo, Samuel B. Pérez, Eduardo Duarte Villa 2, Miguel de Dios Hernández and Guillermo González-Sánchez

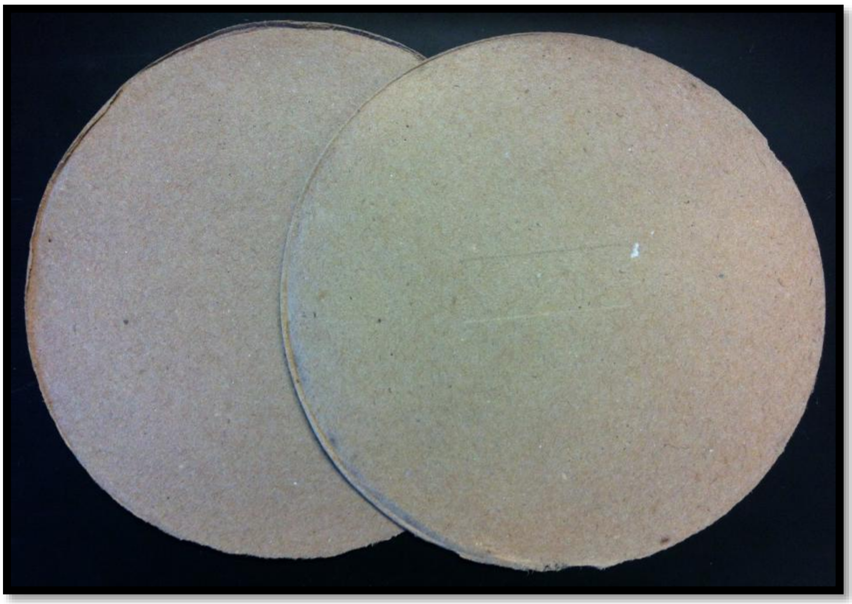

Figure S1. A) Paper with no treatment. (left) **B**) Paper treated with Lacasse, Gallic acid, CPX, and CMC (right).
